# Electro-acupuncture for central obesity: randomized, patient-assessor blinded, sham-controlled clinical trial protocol

**DOI:** 10.1186/s12906-021-03367-2

**Published:** 2021-07-03

**Authors:** Linda L. D. Zhong, Xingyao Wu, Tsz Fung Lam, Ying Ping Wong, Peihua Cao, Emily Yen Wong, Shipping Zhang, Zhaoxiang Bian

**Affiliations:** 1 Hong Kong Chinese Medicine Study Centre, School of Chinese Medicine, Hong Kong Baptist University, 3/F, Jockey Club Chinese Medicine Building, 7 Baptist University Road, Kowloon, Hong Kong SAR, P. R. China; 2 Department of Hepatobiliary Surgery II, Zhujiang Hospital, Southern Medical University, Guangzhou, China; 3 Department of Family Medicine and Primary Care, Li Ka Shing Faculty of Medicine, The University of Hong Kong, Pok Fu Lam, Hong Kong

**Keywords:** Central obesity, Electro-acupuncture, Randomized controlled trial, Efficacy and safety

## Abstract

**Background:**

Obesity is a common medical condition. Among all the classifications of obesity, central obesity is considered to be a significant threat on the health of individuals. Scientific researches have demonstrated that the accumulation of intra-abdominal fat is associated with higher metabolic and cardiovascular disease risks independently from Body Mass Index (BMI). Our previous research found that the combination of electro-acupuncture and auricular acupressure could significantly reduce the body weight and the BMI compared to sham control group.

**Methods/design:**

This is a patient-assessor blinded, randomized, sham-controlled clinical trial on electro-acupuncture for central obesity. One hundred sixty-eight participants with central obesity will be randomly assigned to two groups, which are the acupuncture group and the sham control group. The whole study duration will be 8-week treatment plus 8-week follow up. The primary outcome is the change in waist circumference before and after the treatment. The secondary outcomes include the changes in hip circumference, waist-to-hip circumference ratio, BMI and body fat percentage during the treatment and follow-up.

**Conclusion:**

The trial will evaluate the efficacy and safety of electro-acupuncture for central obesity compared with sham acupuncture. The study may provide the solid evidence of electro-acupuncture on central obesity in Hong Kong.

**Trial registration:**

ClinicalTrials.gov Identifier: NCT03815253,Registered 24 Jan 2019.

**Supplementary Information:**

The online version contains supplementary material available at 10.1186/s12906-021-03367-2.

## Background

According to the latest epidemiological report worldwide, more than 2.0 billion adults are overweight [1]. 39% of adults aged 18 years and over were overweight, and 13% were obese in 2016 [2]. China is the most affected country in the world, with approximately 11.1% for overweight and 7.9% for obesity in children and adolescents aged 6–17 years, and 34.3% for overweight and 16.4% for obesity in adults (≥18 years) [3]. Data from the Hong Kong Population Health Survey by the Department of Health estimate, 29.9% of people aged 15–84 are obese (BMI ≥ 25.0 kg / m^2^),increasing of 1.5 times compared with 2003. And 20.1% are overweight (23.0 kg / m^2^ ≤ BMI < 25.0 kg), increasing of 1.3 times compared with 2003 in Hong Kong [4]. Obesity is a chronic condition in prevalence which is related to metabolic diseases with serious morbidity and mortality [5]. At the same time, obesity and obesity-related diseases will cause high economic burden on the society [6, 7]. Obesity can be divided into two types, subcutaneous fat type or systemic obesity and abdominal obesity or central obesity. The latter one is defined as accumulation of fat in internal organs and mesenteric membrane in the abdominal cavity [8–10].

Central obesity has strong correlation with insulin resistance, dyslipidemia and systemic inflammation, which plays a crucial role in the pathogenesis of certain chronic diseases [11–13]. Abdominal obesity is also a key risk factor of Alzheimer’s, which can change the structure of the brain and cause the decline of brain function eventually leading to occurrence of dementia [14]. People with abdominal obesity could have 10 times higher risk of getting Alzheimer’s disease compared with the normal body weight people [15]. Evidences from the systematic review and meta-analysis indicated that central obesity also increases the risks of colorectal cancer and gastroesophageal cancer [16–18]. Therefore, the management of patients with central obesity is relatively more crucial and important.

Previous studies showed that lifestyle changes include behavioral therapies, diet changes, and physical exercise, which require high self-discipline and need to implement at leaset 6 months or more to achieve significant improvements [19, 20] on body weight control. Pharmaceutical drugs also have effects but may rebound after stopping medication or have side effects on the personal health [21, 22]. Fewer people would choose to have liposuction but the surgery may lead to serious complications. Chinese medicine provides a series of approaches to treat central obesity. Chinese herbal medicine, acupuncture, moxibustion, cupping, either of which or combined with each other is effective in treating abdominal obesity [23–26]. Acupuncture is the most acceptable therapy among TCM interventions in the treatment of overweight and obesity. According to the previous studies, acupuncture can adjust various metabolic functions, improve the fat decomposition, reduce blood triglycerides levels, and thus achieve weight loss [25, 26]. Currently, more evidences have also been demonstrated in the clinical studies and meta-analyses in terms of the effectiveness and safety of electro-acupuncture [26, 27].

Our previous research found that the combination of electro-acupuncture and auricular acupressure could significantly reduce the body weight and the BMI compared to sham control [28]. However, due to the limitation of research scale, the waist circumference had no significant result and objective metabolic parameters were not tested. Waist circumference is a significant indicator of body fat distribution and an important measurement method for central obesity [29, 30]. Therefore, we conduct this clinical study to provide the solid evidence for acupuncture on central obesity.

### Objectives

The objective of this proposed study is to evaluate the efficacy and safety of the electro-acupuncture in the treatment of central obesity compared with sham acupuncture.

## Methods

### Study design

It will be a patient-assessor blinded, randomized, sham-controlled clinical trial on electro-acupuncture for central obesity. Registered TCM practitioners with at least 3 years of clinical experience and will be trained to treat participants in accordance with study protocols. The TCM practitioners will be aware of the allocation of each patient,but the participants and assessor will be blinded to the group allocation.168 participants with central obesity will be recruited from the public through advertisement. Eligible participants will be randomly assigned to two groups. The treatment group (*n* = 84) will receive electro-acupuncture. The control group will receive sham acupuncture (*n* = 84). Appropriate acupuncture frequency is the premise of effective acupuncture [31]. The frequency of acupuncture in western countries is once a week, while that in China is 2–3 times a week [32]. Participants often go to the clinic will lead to commuting problems, which will affect compliance. Frequent acupuncture affects the sensitivity of acupoints. Therefore,participants will be treated twice a week for a total of 8 weeks. Follow-up will be scheduled 8 weeks after the completion of treatments. Every participant will be administered 16 sessions of electro-acupunture in total (Supplement File 1: SPIRIT Checklist).

The primary outcome will be the change in the waist circumference **at the beginning of the study and at the end of the study**. The secondary outcomes will include the changes in hip circumference, waist-to-hip circumference ratio, Body Mass Index (BMI), body fat percentage. All outcomes will be evaluated at 1st,4th,8th,12th,16th session of treatment and the follow up period.

**Fig. 1:**
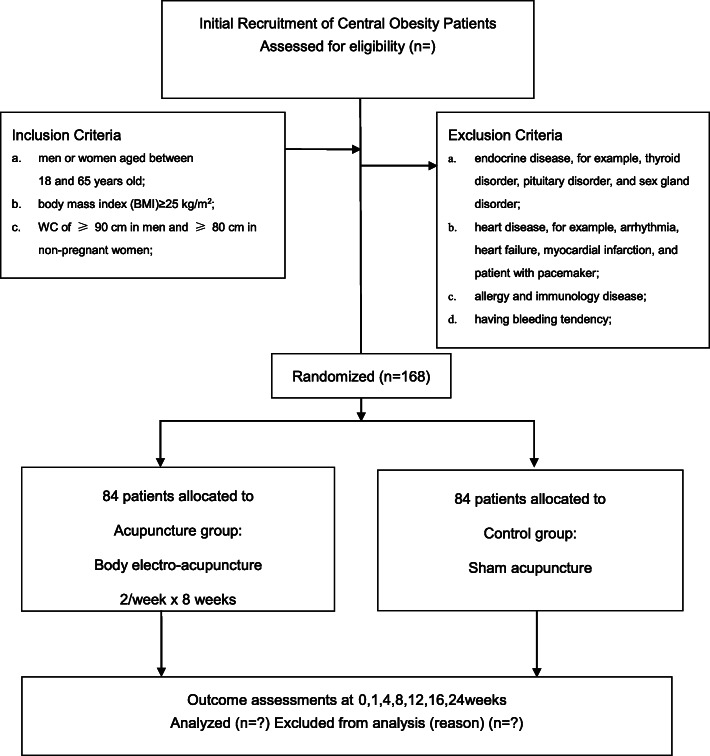
Participant flow chat

The study protocol has been registered in www.clinicaltrials.gov. The flowchart of the trial is shown in Fig. 1.The details of Revised STandards for Reporting Interventions in Clinical Trials of Acupuncture (STRICTA) checklists for items is given in Table 1 [33].

**Table 1:** Checklists for items in STRICTA 2010

Item	Detail
**1.Acupuncture rationale**	**1a) style of acupuncture** Our previous study found that the combination of electroacupuncture and auricular massage significantly reduced weight and BMI compared to sham controls.
	**1b) reasoning for treatment provided, based on clinical** experience, literature sources, and /or consensus methods, with references where appropriate
	**1c) extent to which treatment was varied:** Use standard processing. No difference in treatment between patients
**2.Details of needling**	**2a) number of needle insertions per subject per session (mean and range where relevant):** 14 needles
	**2b) names (or location if no standard name) of points used (uni/bilateral)** Tianshu (ST-25,Daheng (SP-15), Daimai (GB-26), Qihai (CV-6), Zhongwan (CV-12),Zusanli (ST-36),Fenlong (ST 40),Sanyinjiao (SP-6),^.^
	**2c) Depth of insertion, based on a specified unit of measurement, or on a particular tissue level** inserted at a depth of 10–25 mm into the points
	**2d) response sought (e.g. de qi or muscle twitch response):** De qi
	**2e) Needle stimulation (e.g. manual, electrical)** Electrical stimulation with dense-disperse waves with 50 Hz at 100 V will be delivered through electrical acupuncture stimulation instrument (ES-160 6-Channel Programmable Electro-acupuncture) to the abdominal points.
	**2f) needle retention time:** 30mins
	**2 g) Needle type (diameter, length, and manufacturer or material)** Disposable acupuncture needles (verum acupuncture needles asia-med Special No. 16 with 0.30 × 0.30 mm matching the Streitberger sham-needles) *Sham acupuncture:* Streitberger’s non-invasive acupuncture needles (Gauge 8 × 1.2″ / 0.30 × 30 mm)
**3.Treatment regimen**	**3a) number of treatment sessions:** The number of treatment sessions is 16 sessions.
	**3b) frequency and duration of treatment sessions:** The frequency and duration of treatment sessions is twice in a week over 8 consecutive weeks, every session will take 30 min.
**4.Other components of treatment**	**4a) details of other interventions administered to acupuncture group (e.g. moxibustion, cupping, herbs, exercise, lifestyle advice):** Participants be told not to eat other snacks, meat or starchy foods in addition to meals, etc.
	**4b) setting and context of treatment, including instructions to practitioners, and information and explanations to patients:** Hong Kong Baptist University Chinese Medicine Clinics Participants will be informed about acupuncture treatment in the study as follows: “in this study, acupoints for central obesity will be used based on related reports and clinical experience of our investigators.”
**5.Practitioner background**	**5) description of participating acupuncturists (qualification or professional affiliation, years in acupuncture practice, other relevant experience):** Registered TCM Physician with at least 3 years of clinical experience and trained to treat participants in accordance with study protocols.
**6.Control or comparator interventions**	**6a) The primary outcome is the change in waist circumference at the beginning of the study and at the end of the study. The secondary outcomes include** changes in hip circumference, waist-to-hip circumference ratio, Body Mass Index (BMI), body fat percentage, total cholesterol (TC), triglyceride (TG), fasting blood glucose (FBG) during treatment and follow up period.
	**6b)** **-style of acupuncture** **Sham acupuncture** **-number of needle insertions per subjects per session:** 14 sham needles at the same acupoints as the treatment group -Depth of insertion: Needles are only adhered to the skin of insertion. -**needle retention time:** 30 min **-needle type** Streitberger’s non-invasive acupuncture needles (Gauge 8 × 1.2″/ 0.30 × 30 mm) **-frequency and duration of treatment sessions:** 2 sessions per week over 8 consecutive weeks.

### Participants

#### Inclusion criteria

Participants will be included the trial if they meet the following criteria:

1. In the past 3 months, they have not received weight loss treatment by Chinese medicine, conventional medicine or nutritionist.
2. Participans’ aged between 18 and 65 years old;
3. Central obesity, waist circumference of men ≥90 cm, waist circumference of women ≥80 cm;
4. The body mass index (BMI) ≥ 25 kg/m^2^.

#### Exclusion criteria

Participants will be excluded the trial if they meet the following criteria:

1. Having Endocrine system diseases,for example thyroid disorder, pituitary disorder, and sex gland disorder;
2. Having impaired hepatic or renal function;
3. Having heart disease, for example, arrhythmia, heart failure, myocardial infarction, and patient with pacemaker;
4. Pregnant or lactating women;
5. Having bleeding tendency;
6. Having allergy and immunology disease;
7. Having bleeding coagulation disorders;
8. Stroke or otherwise unable to exercise.

### Setting

The study will be conducted in eight Chinese Medicine Clinics, School of Chinese medicine, Hong Kong Baptist University All the participants will sign the consent form and be explained the risks and benefits of the study in details (Supplementary File 2: Patient Consent Form).

To assess nutritional intake, we designed a diet diary that includs food intake and exercise patterns for participants to record daily. Their diary will be reviewed by researchers every follow-up session.

### Recruitment

We will recruit participants through advertisements and TV programs. Screening will be assessed by researchers. Eligible participants will sign the consent form before randomisation.

### Interventions

#### Electro-acupuncture treatment

##### The acupuncture prescription

According to systematic review [25], the highly frequently used acupuncture points in body weight control trials are Zusanli (ST-36), Sanyinjiao (SP-6), Tianshu (ST-25), Fenlong (ST-40), Zhongwan (CV-12), Qihai (CV-6) In addition to the above acupuncture points, based on our principle investigator and co-investigators clinical experience, we will add two acupoints on the abdomen that are effective for central obesity, Daheng (SP-15), Daimai (GB-26). There are 8 acupoints and 14 needling points in total.. The location of each acupuncture points were listed in Table 2.

**Table 2:** The acupuncture points and its Locations

Acupuncture points	Locations
Tianshu (ST-25)	2-in. lateral to the AML level with the umbilicus (CV 8)
Daheng (SP-15)	4-in. lateral to the center of the umbilicus (CV 8) lateral to rectus abdominus
Daimai (GB-26)	Directly below LV 13 at the crossing point of a vertical line through the free end of the 11th rib and a horizontal line through the umbilicus (level with CV 8)
Qihai (CV-6)	Midway between CV 5 and CV 7, 1.5-in. below CV 8 (umbilicus)
Zhongwan (CV-12)	Midway between CV 8 and CV 16, 4 -in. above CV 8 (umbilicus)
Zusanli (ST-36)	3-in. below ST 35, one finger width lateral from the anterior border of the tibia
Fenlong (ST-40)	8-in. below ST 35, one finger width lateral to ST 38, two finger widths lateral to the anterior border of the tibia
Sanyinjiao (SP-6)	3-in. directly above the tip of the medial malleoulus on the posterior border of the tibia

##### Electro-acupuncture

The course of treatment of this clinical experiment is 8 weeks, a total of 16 sessions, twice a week. The needle retention time is 30 min at each session. First, the TCM physician will instruct the participants to lie supine on the treatment bed, exposing the abdomen and legs for disinfection. Then, the TCM physician will use acupuncture needles (verum acupuncture needles asia-med Special No. 16 with 0.30 × 0.30 mm matching the Streitberger sham-needles) to puncture the specified points, a total of 14 points. The insertion depth of each acupoint is about 10–25 mm to achieve Deqi sensation, a feeling of soreness, numbness, heaviness and pressure soreness by patient or a feeling of heavy, tight, astringent, stagnant by TCM physician [34]. Electroacupuncture will then be applied to the abdominal points with 50 Hz densely dispersed waves at 50 V through electric needle stimulator (ES-160 6-Channel Programmable Electro-acupuncture), and the handle of the needle will start to tremble slightly.

##### Sham acupuncture

Streitberger’s non-invasive acupuncture needle (specification 8 × 1.2 in. / 0.30 × 30 mm) will be used at the same acupuncture point in the same stimulation manner but the needles will only adhere to the skin and not be inserted. The validity and credibility of the model have been fully proven. At the same time, the group of the sham acupuncture also will be connected to a electric needle stimulator, but no electrical stimulation in the body, just the stimulator will emit the same beeping sound and flashing light continuously.

##### Auricular acupressure

All the Subjects receive unilateral auricular acupressure at four auricular points including Hunger, Shen men, Spleen and Stomach with Semen Vaccariae (Wang Bu Liu Xing) embedded within adhesive tape in each treatment session. Participants are instructed to repeatedly press the tape with fingertips for 1 min per point, thrice per day. The embedded tape was retained in-situ for 24 h and then the alternate ear would be treated at the next visit.

### Lifestyle intervention

All participants will be advised to take regular number of meals daily and not to intake any snacks. Meals will be comprised of one bowl of rice (210 g) for subjects> 70 kg and two-thirds of a bowl of rice (140 g) for those < 70 kg, with instructions to eat side dishes balanced with the rice [35]. In addition, subjects will be required not to take exercise except for essential activities in their daily work in order to evaluate the sole effect of acupuncture treatment (Supplement File 3: Patient Diet Diary).

### Outcome measures

The primary outcome will be the changes in waist circumference before and after the treatment. Secondary outcomes will include the changes in hip circumference, waist-to-hip circumference ratio, Body Mass Index (BMI), body fat percentage, total cholesterol (TC), triglyceride (TG), fasting blood glucose (FBG) during treatment and follow up period. TC, TG and FBG will be measured before and after the 8-week treatment. Except for the baseline (week 0), these numbers are measured weekly during the treatment period. Body weight, BMI and body fat percentage are measured by body Omron Karada Scan HBF-701. Adverse events of acupuncture treatment will be assessed using the Treatment Emergent Symptom Scale (TESS).

### Randomization assignment

Participants in the two groups will be randomly assigned to the experimental group (real needle) and the control group (sham needle). For randomization, the computer program will pre-generate simple, complete, non-sequential random numbers (in groups of 4) and save them by the lead investigator (PI, LDZ). After confirming that the participant is eligible, the PI will provide the acupuncturist with a random number corresponding to the group assignment. This design is to ensure that clinical assessors and participants are not informed about the distribution.

### Blinding process

This is a patient-assessor blinded sham-controlled clinical trial. The participants will be blinded by using Streitberger’s non-invasive acupuncture needle (specification 8 × 1.2 in. / 0.30 × 30 mm) at the same acupuncture point in the same stimulation manner. Only the TCM practitioners know the allocation of each patient. The assessors, and the statistician performing the data analyses will be blinded to the group allocation throughout the study. After the treatment, the participants will be asked about perceived treatment allocation to evaluate the successful rate of blinding. Only when the lead investigator (PI, LDZ) measurement is critical to patient safety, such as in medical emergencies, can blindness be eliminated on a case-by-case basis.

### Sample size

The calculation of the sample size is based on the measurement of the main results.

Our preliminary clinical study of 72 participants’ results [28] showed that electroacupuncture combined with auricular acupressure decreased from baseline to week 8 by 1.57% (sd = 0.025) in WC, compared with 0.14% (sd. = 0.041) in the sham group). Considering 80% efficacy and 5% alpha (two tails), at least 70 subjects in each group were required to test its importance. Considering a 20% dropout rate, we plan to recruit 84 subjects per group, for a total of 168 subjects. Calculations were performed using PASS 11 software in Caseville, Utah, USA.

### Data processing and analysis

Statistical analysis will be performed using the Social Science Statistics Package (SPSS) for Windows version 23.0. Statistical significance was defined as a two-sided *P* value of < 0.05. Efficacy and safety analyses will be performed in accordance with the intention-to-treat (ITT) principle. Missing values will be estimated by the method carried by the last observation. Baseline characteristics will be reported as mean (SD). Normally distributed continuous variables will be evaluated using the Student’s test, and the non-normal distribution will be evaluated using the non-parametric Mann-Whitney U test to assess baseline differences between the two groups. For categorical variables, chi-square tests or Fisher’s precise tests are used. The analysis of covariance based on ANCOVA was used. ANCOVA was used to compare the treatment groups and subscales, with the treatment group as the model factor and the baseline as the baseline. Changes in covariate scores from baseline to the end of treatment were examined by repeated analysis of variance (ANOVA). The normal distribution data were tested by paired t test, while the non-normal distribution data were tested by Wilcoxon symbol rank test. The paired t test was used to evaluate the normal distribution data, and the Wilcoxon positive and negative rank test was used to evaluate the normal distribution data. The paired t test was used to evaluate the normal distribution data, while the Wilcoxon symbol rank test was used for the non-normal distribution data.

### Data management and confidentiality

Participants’ hard-copy data will be processed by researchers and stored in lockers or encrypted on designated computers. Locker keys and database passwords are kept only by investigators. Seven years after the end of the project, hard copies need to be shredded and soft copies need to be deleted.

## Discussion

The study is a patient-assessor blinded, randomized controlled clinical trial. To date, researches are mainly on treating general obesity but not targeting to reduce abdominal fat. Therefore, we design this clinical trial focusing on the treatment of central obesity and it would be the first of such research on the local Hong Kong Chinese ethic group.

Research on integrated treatments with electro-acupuncture, Chinese Herbal Medicine (CHM), diet plan, physical exercises for central obesity and research on the influence of electroacupuncture to serum glucose, cholesterol, blood pressure or psychological condition such as mood-influenced satiety can be developed with the data from this clinical trial. There are 8 points selected: Tianshu (ST-25), Daheng (SP-15), Daimai (GB-26), Qihai (CV-6), Zhongwan (CV-12), Zusanli (ST-36), Fenlong (ST-40), Sanyinjiao (SP-6) (Table 2). The rationale of choosing these acupoints is based on the scientific laboratory findings and the evidence-based systematic reviews and clinical studies [24–27].

The limitations of this study are that the selection of acupuncture points and the strength of electro stimulation are standardized and utilized for every subject without individual differentiation. We poposed to identify the individual effects of this standardized acupuncture procedure in our further study. Different strength of electro stimulation may also need to be further investigated according to subject’s waist size i.e. the larger waist circumference the stronger electro stimulation. Another limitation is the standardized depth of insertion. The subject’s needling sensation of tingling and numb or “De-qi” which is traditionally the indicator of point reaching may be varied with their body size.

## Conclusion

The results of this study will provide the basis for the effectiveness and safety of electroacupuncture in the treatment of central obesity. Traditional Chinese Medicine (TCM) can use acupuncture to treat central obesity or other problems related to the disease.

## Trial status

The participants are currently being recruited for the present study. We have recruited 57 participants currently on Dec. 22, 2019.

## Supplementary Information

**Additional file 1.** SPIRIT 2013 Checklist.

**Additional file 2.** Patient Consent Form.

**Additional file 3.** Patient Diet Diary.

## Data Availability

Details of this study are available from the corresponding author upon request.

## Abbreviations

BMI: Body mass index
AST: Aspartate aminotransferase
ALT: Alanine transaminase
TC: Total cholesterol
TG: Triglyceride
FBG: Fasting blood glucose
TESS: Treatment emergent symptom scale
SAE: Severity adverse event
ANCOVA: Analysis of covariance
ANOVA: Analysis of variance
AMPK: Activated protein kinase
CHM: Chinese herbal medicine
ITT: Intention-to-treat
CMP: Chinese medicine practitioner
