# Supplementary material for: Electro-acupuncture for central obesity: randomized, patient-assessor blinded, sham-controlled clinical trial protocol
Source: BMC Complement Med Ther. 2021 Jul 3;21:190. doi: 10.1186/s12906-021-03367-2 (PMC8254909; doi:10.1186/s12906-021-03367-2)
Supplement: Supplementary file 2 — Additional file 2. Patient Consent Form. [file 12906_2021_3367_MOESM2_ESM.docx]

**HONG KONG BAPTIST UNIVERSITY**

**ELECTRO-ACUPUNCTURE FOR CENTRAL OBESITY：RANDOMIZED, PATIENT-ASSESSOR BLINDED, SHAM-CONTROLLED CLINICAL TRIA**

**INFORMED CONSENT STATEMENT**

You are invited to participate in a research study.

**Background**

Obesity is an increasingly prevalent chronic condition that is associated with serious morbidity and mortality. In Hong Kong, 36.6% of the population aged 18-64 were classified as overweight or obese (BMI≥23), including 18.8% as obese (BMI≥25) among all the population in 2013. Among all classifications of obesity, central obesity is considered to be the subtype which should pay more attention. Modern researches have demonstrated that the accumulation of intra-abdominal fat is associated with higher metabolic and cardiovascular disease risk independently of BMI. Our previous research found that the combination of electro-acupuncture and auricular acupressure could significantly reduce the body weight and the BMI compared to sham control. However, due to the limitation of research scale, the waist circumference had no significant result and objective metabolic parameters were not tested. Therefore, we plan to conduct this clinical study to serve further and solid evidence for electro-acupuncture on central obesity.

**Study Aim**

The aim of this clinical study is to assess the efficacy and safety of electro-acupuncture compared to sham acupuncture on central obesity.

**Project Title**

Electro-Acupuncture for Central Obesity：Randomized, Patient-Assessor Blinded, Sham-Controlled Clinical Tria

**Study Plan**

This is a single-blind, randomized, sham-controlled trial. 168 participants will be randomly assigned to acupuncture group or control group. Randomization is that your treatment regimen is determined using mathematical and statistical methods, but not subjective methods and you are expected to be not aware which treatment regime you will receive. The duration of the treatment will be 8 weeks with 2 sessions per week and the follow-up period will be 8 weeks. There will 16 sessions of acupuncture treatment within 8 weeks. Each session lasts about 30 min. In addition, blood tests will be arranged at baseline (week 0), end of treatment (week 8). Laboratory variables including total cholesterol (TC), triglyceride (TG) and fasting blood-glucose (FBG) will be tested.

**Risks and Emergency Medical Treatment**

Acupuncture treatment may cause the feeling of soreness, numbness, and heaviness, but, in general, does not cause significant discomfort and severe side effects. However, sometimes mild pain and bruise may occur in acupuncture points. Once any discomforts and unexpected symptoms and side effects occur, we will immediately notify you so that you could make decision whether you continue to participate in the study. Further medical treatment or referral to **Mr. and Mrs. Chan Hon Yin Modern Chinese Medicine Research and Service Centre** will be arranged, if necessary.

**Benefits**

Research leads to many advances in diagnosis and treatment of illness. Taking part in this research may not only benefit to you individually, but also helps to find a treatment for central obesity. However, if positive results could be obtained from this study, your participation is no doubt beneficial to you and other patients in the future. Free charges for acupuncture treatment will be provided to you.

**Responsibilities**

If you agree to participate in this study, you need to provide your personal information, including name, age, address, telephone number, email address and medical history. You will be instructed to eat the same number of meals regularly and not to eat any snacks, meat or wheat flour meal.

**Confidentiality**

All information only for research used and no personal information would be released.

**Compensation and Insurance**

You have no compensation and charged for participating of this study. After the completion of this study, you can still seek for medical treatment in our clinics at your own cost. The study is covered by professional liability insurance policy.

**Contact**

If you have questions at any time about the study or the procedures, you may contact our research team.

Principle investigator: Dr. Zhong Lidan

Telephone number: 3411 6523

Fax number: 3411 2929

If you feel you have not been treated according to the descriptions in this form, or your right as a participant in research have been violated during the course of this project, you may contact the Committee on the Use of Human and Animal Subjects in Teaching and Research of Hong Kong Baptist University.

**Participation**

Your participant in this study is voluntary; you may decline to participate without penalty. If you decide to participate, you may withdraw from the study at any time without penalty and without loss of benefits to which you are otherwise entitled. If you withdraw from the study before data collection is completed your data will be returned to you or destroyed.

**Consent**

I have read and understand the above information. I have received a copy of this form. I agree to participate in this study.

Subject's signature______________________________Date _______________________

Investigator's signature___________________________Date _______________________

**香港浸會大學**

**電針治療中央型肥胖的單盲隨機安慰劑對照臨床研究**

**~研究內容及病人同意聲明書~**

閣下獲邀參加一項有關電針對於中央型肥胖療效的臨床研究

**資料背景**

肥胖目前在世界範圍內的發病率逐年上升，並且由於其可能導致的嚴重疾病日益受到重視。2013年的統計數據顯示，香港18至64歲的人口中約有36.6%超重(BMI≥23)，其中約有18.8%是肥胖人群(BMI≥25)。在所有肥胖的分類中，中央型肥胖是最應被關注的類型之一。現代研究發現，腹部脂肪的堆積是獨立於BMI之外的代謝性疾病和心血管疾病的風險因素。我們之前進行的一項研究證實，相較於安慰劑，電針聯合耳穴貼壓治療可以顯著降低體重和BMI。但是由於研究規模的限制，電針聯合耳穴貼壓治療對於腰圍的改變並不顯著，並且客觀的代謝性指標也未進行檢測。因此，我們計劃開展是項臨床研究，以期提供進一步電針治療中央型肥胖的臨床證據。

**研究目的**

故本次研究目的在於通過隨機臨床研究，評估電針對於中央型肥胖的療效及安全性。

**計劃名稱**

電針治療中央型肥胖的單盲隨機安慰劑對照臨床研究

**研究計劃**

這是一個隨機單盲的研究，168名參試者將會隨機分為真針組或安慰劑組。所謂隨機分組，是用統計數學方法來確定你接受哪一種治療，其原則是，有同等的機會被分配到任何一種治療，但無法預知將被分配到哪一組。真針組、安慰劑組會進行每4週的評估治療及8週的治療，以及之後8週的跟進評估。整個研究為期16週。治療期間，每週將進行兩次針灸治療，總共16次治療，每次大約30分鐘。另外，在研究開始時（0週），治療結束時（8週）會安排2次血液檢查，檢查的實驗室指標包括總膽固醇、甘油三酯及空腹血糖。

**風險評估及緊急醫療操施**

除酸麻脹外﹐一般情況下針刺不會造成明顯不適和嚴重不良反應，但有時會有一些疼痛感和出現皮下瘀腫。如閣下在計劃期間感到不適，我們會即時終止研究，如有需要將轉介往陳漢賢伉儷現代中醫治療研究中心作進一步治療。

**研究效益**

科學研究有助於醫學的發展，雖然不能保證每一參試者都能獲得滿意的治療效果，然而，你的參與無疑對於使用針刺治療中央型肥胖的臨床研究做出了貢獻。本次研究所有的治療均為免費。

**參加者需履行義務**

如閣下同意參加是次研究，你需向研究小組提供個人資料，包括姓名、年齡、地址、聯絡電話和電郵地址等，及有關閣下健康情況的資料，或需出示病歷證明。閣下的日常飲食也會被告知出正餐外不得進食其他的零食、肉食或澱粉質食物等。

**私隱保障**

所有資料只作研究用途，任何個人資均不會對外公佈。

**補償及保險安排**

閣下參與是次的研究是沒有任何費用或額外酬勞的。在完成整個研究後，閣下可自費繼續在本校中醫診所繼續接受診治。本研究已投保專業責任保險，所有參加者均包括在保障範圍內。

**聯絡資料**

閣下對是次的研究有任何查詢，請即與研究小組人員提出，我們非常樂意為你作進一步解釋。

研究總負責人︰鍾麗丹博士

電話︰3411 6523

傳真︰3411 2929

如閣下接受的治療與此同意書中描述的相關內容有出入，或作為研究參試者的權益受到侵害，可聯絡香港浸會大學道德委員會。

**參予條款**

閣下是自願參與是次臨床研究，並擁有隨時退出本研究的權利。閣下拒絶或提前退出參與本研究，是不會對你的醫療護理構成任何損失或懲罰的。如閣下在完成收集所需資料前決定退出本研究，所有已獲得的資料將回發還銷毀。

**病人同意書**

本人已瞭解以上所有內容，持有同意書副本，並自願同意參加本研究。

參加者簽署_________________________________ 日期 _______________________

研究人員簽署________________________________ 日期 _______________________
