# Supplementary material for: Electro-acupuncture for central obesity: randomized, patient-assessor blinded, sham-controlled clinical trial protocol
Source: BMC Complement Med Ther. 2021 Jul 3;21:190. doi: 10.1186/s12906-021-03367-2 (PMC8254909; doi:10.1186/s12906-021-03367-2)

**
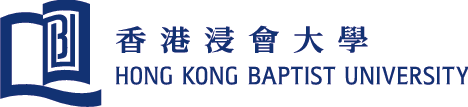
**

**
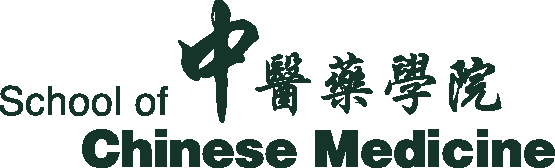
**

**電針治療中央型肥胖**

**的單盲隨機安慰劑對照臨床研究**

**ELECTRO-ACUPUNCTURE FOR CENTRAL OBESITY**

**飲食日誌**

**姓名 name︰ ____________________**

**編號 no.︰ ____________________**

**日期 date︰ ____________________**

飲食參考：

1. 每日用餐定時定量
2. 除正餐外不得進食其他的零食、肉食或澱粉質食物等

Note:

1. To have regular number of meals daily
2. Not to have any snacks

填寫示範 Example **（只需填寫治療前1天的飲食狀況）**

| 第 __1__ 周  (___/___ 至 ___/___) | 第 __1__ 日  ____ / ____ | 第 __2__日  ____ / ____ |
| --- | --- | --- |
|  | 食物與份量  food & amount |  |
| 早餐  Breakfast | 脫脂牛奶1 杯  skimmed milk 1 glass  即溶麥片  instant oatmeal 20g |  |
| 小吃  Light bites | 蘋果 apple 1 個 |  |
| 午餐  Lunch | 牛油多士butter toast 1 片slice  炒蛋 scrambled egg 2 個  雪菜肉絲米粉1碗（米粉100g）  sliced pork vermicelli (carbo ~100g)  奶茶 tea with milk 1杯 cup  白砂糖 white sugar 1茶匙 teaspn |  |
| 小吃  Light bites | 草莓 strawberry 200g  原味餅乾 original digestive 1片 |  |
| 晚餐  Dinner | 煎魚柳 1片  pan fried fish fillet 100g  炒紅蘿蔔 fried carrot 150g  炒西蘭花 fried broccoli 150g  白飯 plain rice ⅔碗（120g） |  |
| 總熱量（calories）  Daily energy intake | （由研究員填寫 by researcher） |  |

| 第 ____ 周  (___/___ 至 ___/___) | | 第 ____ 日  ____ / ____ | | 第 ____ 日  ____ / ____ | | 第 ____ 日  ____ / ____ |
| --- | --- | --- | --- | --- | --- | --- |
|  | | 食物與份量  food & amount | | 食物與份量  food & amount | | 食物與份量  food & amount |
| 早餐  Breakfast | |  | |  | |  |
| 小吃  Light bites | |  | |  | |  |
| 午餐  Lunch | |  | |  | |  |
| 小吃  Light bites | |  | |  | |  |
| 晚餐  Dinner | |  | |  | |  |
| 總熱量（calories） | |  | |  | |  |
| 運動種類  及維持時間 | |  | |  | |  |
| 第 ____ 日  ____ / ____ | 第 ____ 日  ____ / ____ | | 第 ____ 日  ____ / ____ | | 第 ____ 日  ____ / ____ | |
| 食物與份量  food & amount | 食物與份量  food & amount | | 食物與份量  food & amount | | 食物與份量  food & amount | |
|  |  | |  | |  | |
|  |  | |  | |  | |
|  |  | |  | |  | |
|  |  | |  | |  | |
|  |  | |  | |  | |
|  |  | |  | |  | |
|  |  | |  | |  | |
| 第 ____ 周  (___/___ 至 ___/___) | | 第 ____ 日  ____ / ____ | | 第 ____ 日  ____ / ____ | | 第 ____ 日  ____ / ____ |
|  | | 食物與份量  food & amount | | 食物與份量  food & amount | | 食物與份量  food & amount |
| 早餐  Breakfast | |  | |  | |  |
| 小吃  Light bites | |  | |  | |  |
| 午餐  Lunch | |  | |  | |  |
| 小吃  Light bites | |  | |  | |  |
| 晚餐  Dinner | |  | |  | |  |
| 總熱量（calories） | |  | |  | |  |
| 運動種類  及維持時間 | |  | |  | |  |
| 第 ____ 日  ____ / ____ | 第 ____ 日  ____ / ____ | | 第 ____ 日  ____ / ____ | | 第 ____ 日  ____ / ____ | |
| 食物與份量  food & amount | 食物與份量  food & amount | | 食物與份量  food & amount | | 食物與份量  food & amount | |
|  |  | |  | |  | |
|  |  | |  | |  | |
|  |  | |  | |  | |
|  |  | |  | |  | |
|  |  | |  | |  | |
|  |  | |  | |  | |
|  |  | |  | |  | |
| 第 ____ 周  (___/___ 至 ___/___) | | 第 ____ 日  ____ / ____ | | 第 ____ 日  ____ / ____ | | 第 ____ 日  ____ / ____ |
|  | | 食物與份量  food & amount | | 食物與份量  food & amount | | 食物與份量  food & amount |
| 早餐  Breakfast | |  | |  | |  |
| 小吃  Light bites | |  | |  | |  |
| 午餐  Lunch | |  | |  | |  |
| 小吃  Light bites | |  | |  | |  |
| 晚餐  Dinner | |  | |  | |  |
| 總熱量（calories） | |  | |  | |  |
| 運動種類  及維持時間 | |  | |  | |  |
| 第 ____ 日  ____ / ____ | 第 ____ 日  ____ / ____ | | 第 ____ 日  ____ / ____ | | 第 ____ 日  ____ / ____ | |
| 食物與份量  food & amount | 食物與份量  food & amount | | 食物與份量  food & amount | | 食物與份量  food & amount | |
|  |  | |  | |  | |
|  |  | |  | |  | |
|  |  | |  | |  | |
|  |  | |  | |  | |
|  |  | |  | |  | |
|  |  | |  | |  | |
|  |  | |  | |  | |
| 第 ____ 周  (___/___ 至 ___/___) | | 第 ____ 日  ____ / ____ | | 第 ____ 日  ____ / ____ | | 第 ____ 日  ____ / ____ |
|  | | 食物與份量  food & amount | | 食物與份量  food & amount | | 食物與份量  food & amount |
| 早餐  Breakfast | |  | |  | |  |
| 小吃  Light bites | |  | |  | |  |
| 午餐  Lunch | |  | |  | |  |
| 小吃  Light bites | |  | |  | |  |
| 晚餐  Dinner | |  | |  | |  |
| 總熱量（calories） | |  | |  | |  |
| 運動種類  及維持時間 | |  | |  | |  |
| 第 ____ 日  ____ / ____ | 第 ____ 日  ____ / ____ | | 第 ____ 日  ____ / ____ | | 第 ____ 日  ____ / ____ | |
| 食物與份量  food & amount | 食物與份量  food & amount | | 食物與份量  food & amount | | 食物與份量  food & amount | |
|  |  | |  | |  | |
|  |  | |  | |  | |
|  |  | |  | |  | |
|  |  | |  | |  | |
|  |  | |  | |  | |
|  |  | |  | |  | |
|  |  | |  | |  | |
| 第 ____ 周  (___/___ 至 ___/___) | | 第 ____ 日  ____ / ____ | | 第 ____ 日  ____ / ____ | | 第 ____ 日  ____ / ____ |
|  | | 食物與份量  food & amount | | 食物與份量  food & amount | | 食物與份量  food & amount |
| 早餐  Breakfast | |  | |  | |  |
| 小吃  Light bites | |  | |  | |  |
| 午餐  Lunch | |  | |  | |  |
| 小吃  Light bites | |  | |  | |  |
| 晚餐  Dinner | |  | |  | |  |
| 總熱量（calories） | |  | |  | |  |
| 運動種類  及維持時間 | |  | |  | |  |
| 第 ____ 日  ____ / ____ | 第 ____ 日  ____ / ____ | | 第 ____ 日  ____ / ____ | | 第 ____ 日  ____ / ____ | |
| 食物與份量  food & amount | 食物與份量  food & amount | | 食物與份量  food & amount | | 食物與份量  food & amount | |
|  |  | |  | |  | |
|  |  | |  | |  | |
|  |  | |  | |  | |
|  |  | |  | |  | |
|  |  | |  | |  | |
|  |  | |  | |  | |
|  |  | |  | |  | |
| 第 ____ 周  (___/___ 至 ___/___) | | 第 ____ 日  ____ / ____ | | 第 ____ 日  ____ / ____ | | 第 ____ 日  ____ / ____ |
|  | | 食物與份量  food & amount | | 食物與份量  food & amount | | 食物與份量  food & amount |
| 早餐  Breakfast | |  | |  | |  |
| 小吃  Light bites | |  | |  | |  |
| 午餐  Lunch | |  | |  | |  |
| 小吃  Light bites | |  | |  | |  |
| 晚餐  Dinner | |  | |  | |  |
| 總熱量（calories） | |  | |  | |  |
| 運動種類  及維持時間 | |  | |  | |  |
| 第 ____ 日  ____ / ____ | 第 ____ 日  ____ / ____ | | 第 ____ 日  ____ / ____ | | 第 ____ 日  ____ / ____ | |
| 食物與份量  food & amount | 食物與份量  food & amount | | 食物與份量  food & amount | | 食物與份量  food & amount | |
|  |  | |  | |  | |
|  |  | |  | |  | |
|  |  | |  | |  | |
|  |  | |  | |  | |
|  |  | |  | |  | |
|  |  | |  | |  | |
|  |  | |  | |  | |
| 第 ____ 周  (___/___ 至 ___/___) | | 第 ____ 日  ____ / ____ | | 第 ____ 日  ____ / ____ | | 第 ____ 日  ____ / ____ |
|  | | 食物與份量  food & amount | | 食物與份量  food & amount | | 食物與份量  food & amount |
| 早餐  Breakfast | |  | |  | |  |
| 小吃  Light bites | |  | |  | |  |
| 午餐  Lunch | |  | |  | |  |
| 小吃  Light bites | |  | |  | |  |
| 晚餐  Dinner | |  | |  | |  |
| 總熱量（calories） | |  | |  | |  |
| 運動種類  及維持時間 | |  | |  | |  |
| 第 ____ 日  ____ / ____ | 第 ____ 日  ____ / ____ | | 第 ____ 日  ____ / ____ | | 第 ____ 日  ____ / ____ | |
| 食物與份量  food & amount | 食物與份量  food & amount | | 食物與份量  food & amount | | 食物與份量  food & amount | |
|  |  | |  | |  | |
|  |  | |  | |  | |
|  |  | |  | |  | |
|  |  | |  | |  | |
|  |  | |  | |  | |
|  |  | |  | |  | |
|  |  | |  | |  | |
| 第 ____ 周  (___/___ 至 ___/___) | | 第 ____ 日  ____ / ____ | | 第 ____ 日  ____ / ____ | | 第 ____ 日  ____ / ____ |
|  | | 食物與份量  food & amount | | 食物與份量  food & amount | | 食物與份量  food & amount |
| 早餐  Breakfast | |  | |  | |  |
| 小吃  Light bites | |  | |  | |  |
| 午餐  Lunch | |  | |  | |  |
| 小吃  Light bites | |  | |  | |  |
| 晚餐  Dinner | |  | |  | |  |
| 總熱量（calories） | |  | |  | |  |
| 運動種類  及維持時間 | |  | |  | |  |
| 第 ____ 日  ____ / ____ | 第 ____ 日  ____ / ____ | | 第 ____ 日  ____ / ____ | | 第 ____ 日  ____ / ____ | |
| 食物與份量  food & amount | 食物與份量  food & amount | | 食物與份量  food & amount | | 食物與份量  food & amount | |
|  |  | |  | |  | |
|  |  | |  | |  | |
|  |  | |  | |  | |
|  |  | |  | |  | |
|  |  | |  | |  | |
|  |  | |  | |  | |
|  |  | |  | |  | |

**附頁 Annex:**

**______________________________________________________________________________________________________________________________________________________________________________________________________________________________________________________________________________________________________________________________________________________________________________________________________________________________________________________________________________________________________________________________________________________________________________________________________________________________________________________________________________________________________________________________________________________________________________________________________________________________________________________________________________________________________________________________________________**

**請於覆診時將日誌交回研究小組**

**多謝合作**

Please bring the Diet Diary for follow-up consultations

| 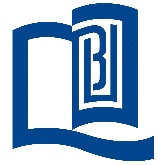 | 香港浸會大學‒賽馬會中醫疾病預防與健康管理中心  地址︰九龍彌敦道771-775號栢宜中心3樓全層 |
| --- | --- |
|  | 香港浸會大學港島魏克強中醫專科診所  地址︰香港干諾道中133號誠信大廈17樓 |
|  | 香港浸會大學中醫專科診療中心  地址︰九龍尖沙咀彌敦道136A號尖沙咀街坊福利會大樓3樓 |
|  | 聯絡電話 tel︰ 3411 6501 |
|  | 電子郵箱 email︰weightcontrol@project.hkbu.edu.hk |


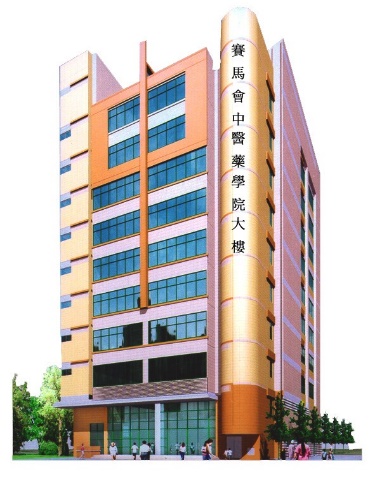

Supplement: Supplementary file 3 — Additional file 3. Patient Diet Diary. [file 12906_2021_3367_MOESM3_ESM.docx]
